# *Fusarium verticillioides* seed inoculation modifies maize cell wall responses to subsequent *Sesamia nonagrioides* infestation

**DOI:** 10.1111/plb.70284

**Published:** 2026-09-10

**Authors:** N. Gesteiro, A. Butrón, P. Lobagueira, X. C. Souto, R. A. Malvar, R. Santiago

**Affiliations:** ^1^ Misión Biológica de Galicia, Sede de Pontevedra (CSIC) Pontevedra Spain; ^2^ As Lagoas Marcosende, Environmental Agro‐biology: Soils and Plants Quality (UVIGO), Associated Unit with MBG (CSIC) University of Vigo Vigo Spain

**Keywords:** borers, cell‐wall, *Fusarium verticillioides*, inducible defences, lignin, maize, resistance

## Abstract

- Maize resistance to stem borers and fungal infection is strongly influenced by cell wall composition, but little is known about how prior fungal seed colonization affects subsequent insect‐induced cell wall responses. Therefore, this study evaluated whether seed inoculation with *Fusarium verticillioides* modifies constitutive and induced cell wall defences against *Sesamia nonagrioides* infestation in maize.
- Seven maize inbred lines were grown under four treatments combining the presence or absence of *F. verticillioides* seed inoculation and *S. nonagrioides* infestation. Tunnel length, lignin content and composition, and cell wall‐bound hydroxycinnamates were quantified in stem pith tissue.
- Treatment effects were mainly detected in lignin composition and hydroxycinnamate accumulation, whereas total Klason lignin and p‐coumaric acid were not significantly altered. In non‐inoculated plants, *S*. *nonagrioides* infestation reduced syringyl lignin subunits (S), ferulic acid and diferulate contents. In contrast, in plants derived from *F. verticillioides*‐inoculated seeds, insect infestation increased ferulic acid and diferulates and shifted lignin composition, reducing S and the syringyl/guaiacyl ratio (S/G) while increasing guaiacyl lignin subunits (G). These responses were strongly genotype‐dependent. A multiple regression model indicated that constitutive levels of S, ferulic acid, p‐coumaric acid and diferulate 8‐O‐4, together with induced changes in G, explained most of the variation in relative tunnel length.
- Overall, these results show that maize resistance to *S*. *nonagrioides* involves both constitutive cell wall traits and inducible cell wall remodelling, and that prior seed colonization by *F*. *verticillioides* can modify the direction and magnitude of these responses, highlighting the importance of stress history and genotype in maize defence.

## Graphical abstract

Prior seed inoculation with *Fusarium verticillioides* altered the maize cell wall response to *Sesamia nonagrioides* attack. The combined biotic interaction promoted specific changes in lignin composition and hydroxycinnamate accumulation, suggesting that stress history modulates inducible structural defences.

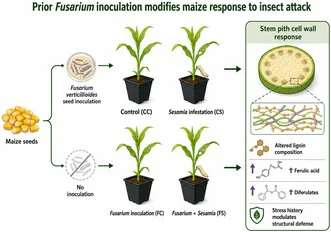

## INTRODUCTION

*Sesamia nonagrioides* Lefebvre (Lepidoptera: Noctuidae), the Mediterranean corn borer (MCB), is considered to be one of the most significant pests of maize in Mediterranean regions, where it has demonstrated to cause substantial economic losses due to the severe damage that larvae inflict on maize stems (Malvar *et al*. 2002). The larvae exhibit a marked preference for consuming the stem pith tissues, resulting in tunnel damage. This damage reduces the flow of nutrients to the grains, thereby causing a reduction in yield, and also compromises the structural integrity of the plants, rendering them more vulnerable to additional stresses. This significant impact has led to growing interest in research on the defence mechanisms of maize against *Sesamia nonagrioides* attack, with the aim of developing effective strategies to mitigate its adverse effects (Kaçar *et al*. 2023).

Cell wall hydroxycinnamates play a pivotal role in plant resistance, acting both as antimicrobial agents that inhibit parasite proliferation and as active cell wall reinforcers (Santiago *et al*. 2005, 2006a, 2013a; Santiago & Malvar 2010). Their structural function, especially when bound to the cell wall, has been extensively studied, highlighting their contribution to resistance against *Sesamia nonagrioides*. Increases in the concentration of *p*‐coumaric acid (*p*CA) and diferulates have been shown to correlate with higher levels of resistance to *Sesamia nonagrioides*, therefore underscoring the importance of these compounds in defence (Santiago *et al*. 2008, 2016; Barros‐Rios *et al*. 2011, 2015). Furthermore, cell wall‐bound *p*CA is considered a key indicator of the lignification process: cell wall‐bound *p*CA is mostly esterified to lignin S subunits. This has led to the hypothesis that both lignin content and composition could be responsible for the observed correlation between *p*CA and resistance to *Sesamia nonagrioides* (Gesteiro *et al*. 2021).

In maize, plants use both constitutive and induced defences to protect themselves from stem borers (Rodríguez *et al*. 2021; Guo *et al*. 2023). Recent evidence has further emphasized that changes in cell wall composition are an active component of plant defence against herbivorous insects, supporting the idea that cell wall remodelling contributes to inducible resistance responses rather than acting only as a constitutive physical barrier (Perez‐Alonso *et al*. 2025). Although the study of resistance to maize stem borers has usually focused on constitutive defences, evidence suggests that induced defences incur reduced costs in terms of resource allocation (Cardinal & Lee 2005; Santiago *et al*. 2006a, 2006b, 2011; Howe & Jander 2008). These induced defences, such as enhanced antibiosis against larvae after stem feeding, may depend on genotype and length of the period of attack (Santiago *et al*. 2017; Rodríguez *et al*. 2021). This highlights the intricacy of defensive responses, which are modulated by the capacity of plants to reprogram their metabolism in response to damage and may result in increased susceptibility or resistance depending on genotype (Schuman & Baldwin 2016).

On the other hand, in temperate regions, the pest effects on the maize plants are further complicated by the frequent infection of maize seeds by the fungus *Fusarium verticillioides* (Wilke *et al*. 2007; Santiago *et al*. 2020). This microorganism, known for its ability to cause Fusarium ear rot disease (FER) and to contaminate kernels with fumonisins under specific environmental and genotypic circumstances, has also been observed as an asymptomatic endophyte (Bacon *et al*. 2008; Santiago *et al*. 2015). Endophytes colonize internal plant tissues without inducing disease symptoms and, in certain instances, can promote host growth and health (Ali *et al*. 2017). In the case of *Fusarium verticillioides*, its ability to promote plant growth, induce defence responses such as lignin deposition, and interfere with infection by other fungi has been previously demonstrated (Yates *et al*. 2005; Lee *et al*. 2009; Rodríguez Estrada *et al*. 2012; Blacutt *et al*. 2018; Gesteiro *et al*. 2024). However, only one previous study has investigated how seed colonization by *Fusarium verticillioides* affects maize defence against *Sesamia nonagrioides*, observing that metabolites and defence pathways are favourably modified in *Fusarium verticillioides*‐resistant genotypes, but not in susceptible ones (Gesteiro *et al*. 2024). More recently, the interaction between *Sesamia nonagrioides* infestation and *Fusarium verticillioides* seed inoculation was also shown to alter physiological indicators in maize stem pith, further supporting the need to consider both biotic agents together rather than as isolated stresses (Fernández‐Descalzo *et al*. 2025).

Although maize cell wall components have been widely associated with resistance to stem borers and fungal pathogens, most studies have considered these stresses separately or have focused on constitutive defences. The novelty of the present work lies in evaluating a sequential biotic scenario in which seed‐associated *Fusarium verticillioides* precedes *Sesamia nonagrioides* infestation, allowing us to determine whether prior fungal colonization modifies the inducible cell wall response to insect attack. This approach provides insight into how stress history shapes maize structural defences under a more realistic multiple‐stress context.

Therefore, the innovative objectives of the present study were: (i) to determine whether seed inoculation with *Fusarium verticillioides* and subsequent infestation by the borer *Sesamia nonagrioides* interferes with the cell wall strengthening, either independently or as a result of the interaction between both treatments; and (ii) to identify the constitutive and/or induced cell wall reinforcement compounds that are most relevant to maize resistance against *Sesamia nonagrioides*.

## MATERIALS AND METHODS

### Plant material and experimental design

Seven inbred lines previously evaluated by Santiago *et al*. (2013b) for resistance to FER were used, including three resistant lines (A509, A630 and EP77) and four susceptible lines (A239, EP125, EP42 and PB130). These lines were subjected to four biotic treatments: seed inoculation with *Fusarium verticillioides* without subsequent infestation with *Sesamia nonagrioides* (FC), seed inoculation with *Fusarium verticillioides* followed by infestation with *Sesamia nonagrioides* (FS), seeds not inoculated with *Fusarium verticillioides* and not infested with *Sesamia nonagrioides* (CC), and seeds not inoculated with *Fusarium verticillioides* but infested with *Sesamia nonagrioides* (CS) (Fig. 1).

**Fig. 1:**
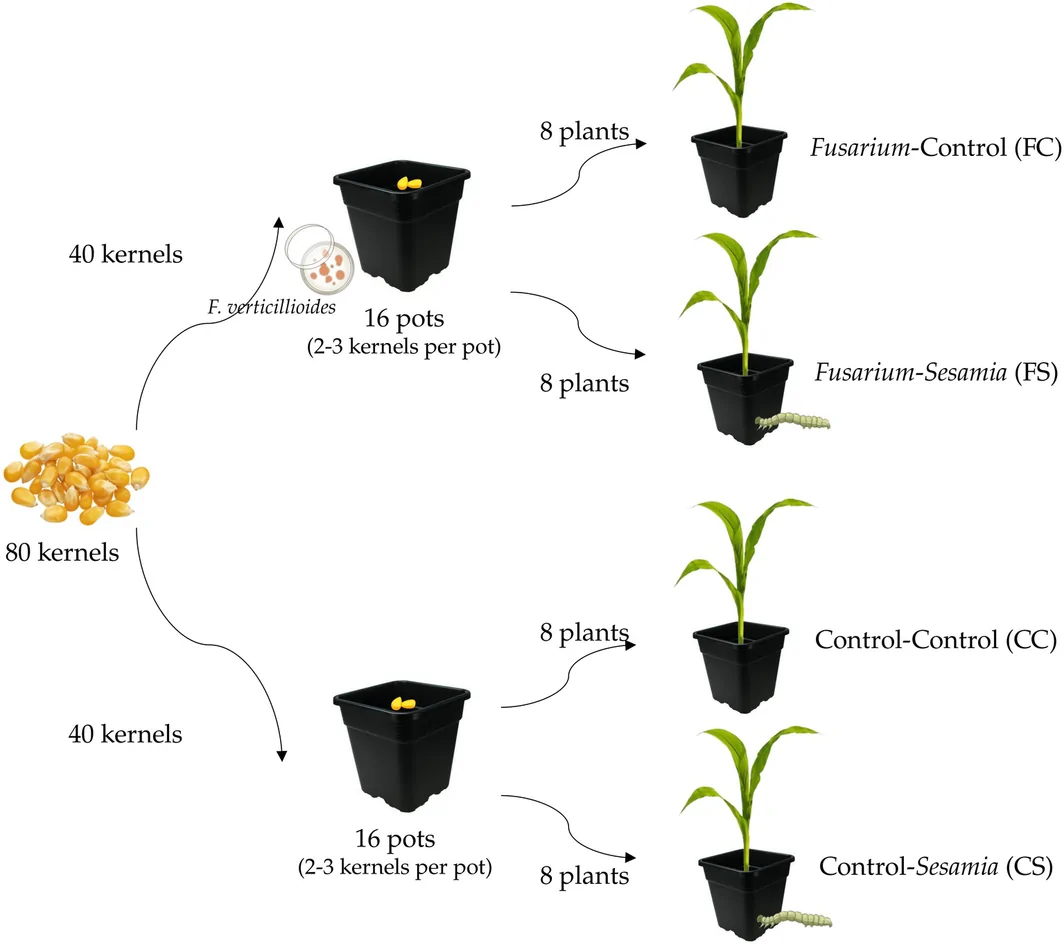
Experimental design to evaluate the response to *Fusarium verticillioides* /*Sesamia nonagrioides* of seven maize inbred lines.

Eighty seeds of each genotype were disinfected following the protocol described by Gesteiro *et al*. (2024), which included immersion in 5.25% sodium hypochlorite, rinsing with sterile distilled water, imbibition in fresh water for 4 h at 25°C and heat treatment at 60°C for 5 min. After disinfection, seeds from each genotype were divided into two groups of 40 seeds. One group was sown directly as non‐inoculated controls, whereas the other group was inoculated by dipping the seeds in a suspension of *Fusarium verticillioides* (10^6^ spores/ml) for 3 min at room temperature before sowing. For each genotype, the 40 non‐inoculated seeds and the 40 inoculated seeds were sown in 16 pots, with 2–3 seeds per pot. At the five‐ to six‐leaf stage (V5–V6), seedlings were thinned to one plant per pot.

Plants were grown under greenhouse conditions at the Misión Biológica de Galicia (CSIC, Pontevedra, Spain), under natural photoperiod and standard greenhouse management. At the tasselling stage, eight plants per genotype–inoculation combination were infested with two *Sesamia nonagrioides* neonate larvae per plant (CS and FS treatments), while another eight plants were kept uninfested (CC and FC treatments). The larvae were obtained from eggs produced by a laboratory colony maintained at the Misión Biológica de Galicia (CSIC), where insects were reared under controlled conditions. Infested and non‐infested plants were placed in separate, fully netted greenhouse sectors to prevent larval movement between treatments while maintaining the same general greenhouse conditions. Further experimental details are available in Gesteiro *et al*. (2024).

Thirty days after infestation, the upper half of the first internode below the ear was collected from six individual plants per treatment and genotype. To ensure sufficient tissue for analysis, samples from two plants were pooled, resulting in three biological replicates per treatment‐genotype combination. The stem rind was removed, and the pith samples were frozen at −80°C, freeze‐dried and ground using a 0.75 mm mesh for subsequent analysis.

### Cell‐wall bound phenolics

#### Lignin content and composition

Lignin content was determined from 50 mg of ground tissue using a modified Klason lignin protocol (Dence 1992), as previously described by Gesteiro *et al*. (2021). Lignin composition was assessed via nitrobenzene oxidation (Pomar *et al*. 2002). The resulting degradation products were analysed by HPLC using a Waters 2690 HPLC system equipped with a diode array detector and a YMC ODS‐AM column (100 × 2 mm, 3 μm). The mobile phase consisted of acetonitrile (A) and 0.05% trifluoroacetic acid in water (B), with a programmed gradient and a flow rate of 0.3 mL min^−1^. Detection was carried out at 280 nm. Degradation products were quantified using standards for p‐hydroxybenzaldehyde (H unit), vanillin (G unit) and syringaldehyde (S unit). Subunit percentages were calculated based on total peak areas.

#### Hydroxycinnamates esterified to the cell wall

Quantification of cell wall‐bound hydroxycinnamates included *p*CA and ferulate (FA) monomers, as well as diferulates (DFAs): DFA 8–5 (sum of the linear and benzofuran forms), DFA 8‐*O*‐4, and DFA 5–5. Total diferulate content (DFAT) was calculated as the sum of these three isomers. For analysis, 0.5 g of ground tissue per sample was used, following the updated method of Santiago *et al*. (2018). Hydroxycinnamates were analysed by high‐performance liquid chromatography (HPLC) under the same chromatographic conditions described above for lignin composition, with detection at 325 nm. Standard recovery was 96–98%.

Retention times were determined using commercial standards (*p*CA and FA, Sigma‐Aldrich) and DFA 5–5 synthesized by Dr John Ralph's group (University of Wisconsin). The following retention times were obtained: *p*CA = 5.34 min, FA = 6.05 min, DFA 8–5 (linear) = 6.60 min, DFA 5–5 = 7.66 min, DFA 8‐*O*‐4 = 8.51 min, and DFA 8–5 (benzofuran) = 8.72 min. The identities of the FA dimerics were confirmed by comparison with the authentic 5–5 standard or with previously published retention times and UV spectra. External quantification was performed using calibration curves with a detection threshold of 2 μg/mL.

#### Statistical analysis

An analysis of variance (ANOVA) for tunnel length and cell wall components was performed using the general linear model (GLM) procedure of SAS (SAS Institute Inc. 2008), considering *Fusarium verticillioides* treatment [inoculation (FC, FS) *versus* no inoculation (CC, CS)], *Sesamia nonagrioides* treatment [infestation (FS, CS) *versus* no infestation (FC, CC)], genotype, and all interactions among these factors as fixed effects. A combined ANOVA was also performed, considering the four treatments (CC, CS, FC, FS) as a fixed treatment effect, along with the effect of genotype and the treatment × genotype interaction. Subsequently, separate ANOVAs were conducted within each *Fusarium verticillioides* treatment: inoculated (FC *versus* FS) and non‐inoculated (CC *versus* CS) to evaluate the response of maize inbreds to *Sesamia nonagrioides* attack. Finally, an ANOVA under no *Sesamia nonagrioides* infestation (FC *versus* CC) was performed to determine whether seed inoculation with *Fusarium verticillioides* alone could modify cell wall components. The normality of residuals was assessed for all models; percentage data were analysed after checking that the residuals did not show substantial departures from normality, and no additional transformation was applied. Genotype means were compared using Fisher's protected least significant difference (LSD).

In addition, we investigated whether the relative tunnel length observed in the FS and CS treatments could be explained by cell wall components. Two types of explanatory variables were considered: (i) constitutive concentrations, defined as the baseline levels of each cell wall compound under control conditions (*i.e*., FC values were used to predict tunnel length in FS, and CC values to predict tunnel length in CS); and (ii) induced concentrations, calculated as the difference between treatments and their respective controls (FS − FC for tunnel length in FS, and CS − CC for tunnel length in CS). These induced values represent the change in compound concentration triggered by *Sesamia nonagrioides* infestation. Both constitutive and induced variables for the analysed compounds—Klason lignin (KL), S, G and H subunits, *p*CA, FA, individual diferulates (DFA 5–5, DFA 8‐*O*‐4, DFA 8–5), and total diferulates—were standardized and included in a multiple regression model to assess their contribution to variation in relative tunnel length. The PROC REG procedure of SAS was applied using a stepwise selection approach, and only variables with a *P*‐value <0.15 were retained in the model.

## RESULTS

The combined analysis including all treatments provided a general overview of the effects of maize genotype, *Sesamia nonagrioides* infestation and *Fusarium verticillioides* seed inoculation on maize cell wall composition. Overall, maize genotype significantly affected all parameters and *Sesamia nonagrioides* infestation significantly affected lignin composition, particularly the relative proportions of S and G subunits and the S/G ratio (Table 1). In contrast, *Fusarium verticillioides* inoculation primarily affected the accumulation of esterified hydroxycinnamates, showing significant effects on all esterified hydroxycinnamates analysed (Table 2).

**Table 1:** Mean squares of the analysis of variance of seven maize inbreds evaluated for Klason lignin (KL) content and percentages of syringyl (S), guaiacyl (G) and *p*‐hydroxyphenyl (H) subunits and syringyl/guaiacyl (S/G) ratio, in stem pith under two *Sesamia nonagrioides* treatments (infestation *versus* no infestation) and two inoculation treatments (seed inoculation with *Fusarium verticillioides versus* no inoculation), resulting in four treatment combinations: (i) control (CC); (ii) infested with *Sesamia nonagrioides* (CS); (iii) inoculated with *Fusarium verticillioides* without subsequent *Sesamia nonagrioides* infestation (FC); and (iv) inoculated with *Fusarium verticillioides* and subsequently infested with *Sesamia nonagrioides* (FS).

	df^a^	KL	S	G	H	S/G
Fusarium	1	29.19	3.51	0.38	3.55	3.6E‐03
Sesamia	1	0.22	60.3^c^	70.83^c^	2.32E‐04	0.27^c^
Fusarium*Sesamia	1	1.97	0.22	1.41	0.86	3.69E‐03
Genotype	6	68.50^c^	98.69^c^	212.23^c^	53.05^c^	0.42^c^
Fusarium*Genotype	6	10.63	1.42	22.14^b^	17.16^c^	2.95E‐02
Sesamia*Genotype	6	67.44^c^	38.78^c^	19.16^b^	24.66^c^	7.79E‐02^c^
Fusarium*Sesamia*Genotype	6	28.01	28.32^c^	15.80	9.04^c^	7.10E‐02^b^
Error	50	7.79	5.37	7.84	1.19	2.24E‐02

All factors and interactions were considered fixed effects. The residual was used as the error term for all tested effects. Mean squares (MS) are presented.

^a^ Degrees of freedom.

^b^ Significant at the probability level of 0.05.

^c^ Significant at the probability level of 0.01.

**Table 2:** Mean squares of the analysis of variance of seven maize inbreds evaluated for esterified hydroxycinnamates content in the cell wall, in stem pith under two *Sesamia nonagrioides* treatments (infestation *versus* no infestation) and two inoculation treatments (seed inoculation with *Fusarium verticillioides versus* no inoculation), resulting in four treatment combinations: (i) control (CC); (ii) infested with *Sesamia nonagrioides* (CS); (iii) inoculated with *Fusarium verticillioides* without subsequent *Sesamia nonagrioides* infestation (FC); and (iv) inoculated with *Fusarium verticillioides* and subsequently infested with *Sesamia nonagrioides* (FS).

	df^a^	*p*‐coumarate	ferulate	DFA 5–5	DFA 8‐*O*‐4	DFA 8–5	DFAT
Fusarium	1	4.29^b^	1.14^c^	2.77E‐03^c^	7.62E‐03^b^	8.93E‐03^c^	5.58E‐02^c^
Sesamia	1	3.91	7.85E‐02	5.23E‐05	6.24E‐03^b^	1.13E‐03	1.32E‐02
Fusarium*Sesamia	1	1.66E‐03	1.07^c^	3.00E‐03^c^	0.18^c^	3.79E‐02^c^	0.45^c^
Genotype	6	56.60^c^	1.99^c^	3.21E‐03^c^	2.30E‐02^c^	2.11E‐02^c^	7.58E‐02^c^
Fusarium*Genotype	6	4.70^c^	0.13^c^	5.34E‐04^c^	1.52E‐03	2.11E‐03^b^	6.03E‐03
Sesamia*Genotype	6	1.51	0.10^b^	1.04E‐03^c^	2.60E‐03	3.54E‐03^c^	1.71E‐02^c^
Fusarium*Sesamia*Genotype	6	7.20^c^	0.14^c^	8.82E‐04^c^	3.02E‐02^c^	1.12E‐02^c^	8.94E‐02^c^
Error	50	1.03	4.04E‐02	1.64E‐04	1.15E‐03	8.90E‐04	4.27E‐03

All factors and interactions were considered fixed effects. The residual was used as the error term for all tested effects. Mean squares (MS) are presented.

^a^ Degrees of freedom.

^b^ Significant at the probability level of 0.05.

^c^ Significant at the probability level of 0.01.

Significant genotype × treatment interactions, including three‐way interactions (*Fusarium* × *Sesamia* × Genotype), revealed that maize responses to insect infestation and fungal inoculation were strongly dependent on genetic background. In addition, *Sesamia nonagrioides* treatment effects on hydroxycinnamate content would depend on the presence of *Fusarium verticillioides* in the seed, as *Sesamia nonagrioides* treatment had only significant effects on DFA 8‐*O*‐4 content, but the interaction *Sesamia* × *Fusarium* was significant for all hydroxycinnamates, except *p*CA (Table 2). Therefore, a new ANOVA analysis was done to get insight into the *Sesamia* × *Fusarium* interaction. All sources of variation were considered fixed: treatments (combinations of *Sesamia nonagrioides* and *Fusarium verticillioides* treatments), genotypes and treatment × genotype.

In general, the treatment significantly affected lignin composition but had no effect on total Klason lignin content (Table 3). Significant differences were detected between treatments in the relative proportions of the S and G subunits, as well as in the S/G ratio, whereas the contribution of the H subunit was not significantly altered by the treatment.

**Table 3:** Mean squares of the analysis of variance of seven maize inbreds evaluated for Klason lignin (KL) content and percentages of syringyl (S), guaiacyl (G), p‐hydroxyphenyl (H) subunits and syringyl/guaiacyl (S/G) ratio in stem pith under four treatments: control (CC), *Sesamia nonagrioides* infestation (CS), *Fusarium verticillioides* inoculation (FC) and plants inoculated with *Fusarium verticillioides* and subsequently infested with *Sesamia nonagrioides* (FS).

	df^a^	KL	S	G	H	S/G
Treatment	3	14.74	21.79^c^	25.81^b^	3.33	0.10^c^
Genotype	6	68.48^c^	98.70^c^	218.58^c^	59.85^c^	0.42^c^
Treatment*Genotype	18	37.78^c^	24.55^c^	17.75^b^	17.57^c^	0.06^c^
Error	52	7.51	5.17	8.14	2.00	0.02

All factors and interactions were considered fixed effects. The residual was used as the error term for all tested effects. Mean squares (MS) are presented.

^a^ Degrees of freedom.

^b^ Significant at the probability level of 0.05.

^c^ Significant at the probability level of 0.01.

Mean comparisons showed that *Sesamia nonagrioides* infestation induced different changes on lignin composition depending on the presence/absence of *Fusarium verticillioides* (Fig. 2). In non‐inoculated plants, *Sesamia nonagrioides* infestation significantly reduced the percentage of S subunits, whereas, in plants grown from seeds previously inoculated with *Fusarium verticillioides*, infestation significantly reduced S and increased the percentage of G subunits, leading to a shift in the S/G ratio (Fig. 2). These results indicate that, although seed colonization by itself did no change lignin content or composition, modulated the lignin compositional response to insect attack. However, this modulation is genotype‐dependent, as genotype × treatment interactions were significant for all lignin traits (Table 3). The study of the responses of each inbred on lignin parameters to *Fusarium verticillioides* (FC *versus* CC), *Sesamia nonagrioides* without previous infection by *Fusarium verticillioides* (CS *versus* CC) and to *Sesamia nonagrioides* on plants previously infected by *Fusarium verticillioides* (FS *versus* FC) are shown in Table S1 and Fig. S1.

**Fig. 2:**
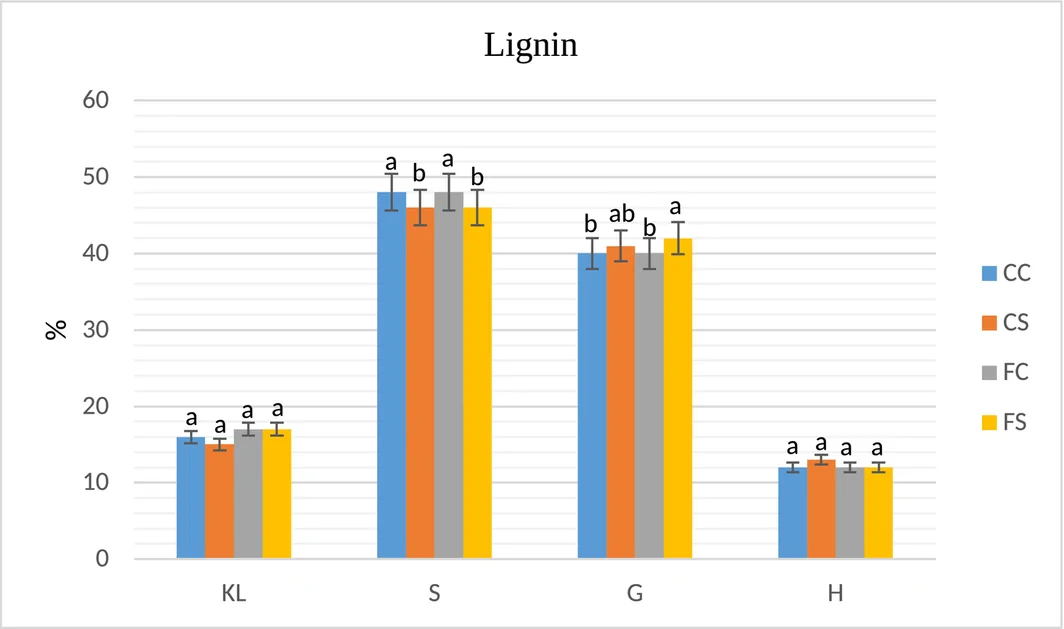
Klason lignin (KL) content and the percentage contribution of syringyl (S), guaiacyl (G) and p‐hydroxyphenyl (H) subunits in the stem pith of seven maize inbred lines evaluated under four treatments: control (CC), infestation with *Sesamia nonagrioides* (CS), inoculation with *Fusarium verticillioides* (FC) and plants inoculated with *Fusarium verticillioides* and subsequently infested with *Sesamia nonagrioides* (FS). Vertical bars represent standard errors. Different letters indicate significant differences among treatments for each trait at the 0.05 probability level.

The effects of the treatment on esterified hydroxycinnamates were more pronounced than those observed for lignin content (Table 4). Significant differences between treatments were detected for FA, all diferulate isomers (DFA 5–5, DFA 8‐*O*‐4, DFA 8–5), and total diferulates, whereas *p*CA content was not significantly affected.

**Table 4:** Mean squares of the analysis of variance of seven maize inbreds evaluated for esterified hydroxycinnamates content in the cell wall, in stem pith under four treatments: control (CC), *Sesamia nonagrioides* infestation (CS), *Fusarium verticillioides* inoculation (FC) and plants inoculated with *Fusarium verticillioides* and subsequently infested with *Sesamia nonagrioides* (FS).

	df^a^	*p*‐coumarate	ferulate	DFA 5–5	DFA 8‐*O*‐4	DFA 8–5	DFAT
Treatments	3	2.81	0.77	1.8E‐3	0.07	0.02	0.18^b^
Genotype	6	62.62	2.25	3.7E‐3	0.02	0.02	0.09
Treat*Genotype	18	4.50	0.13^b^	8.1–4	0.01	5.4E‐3	0.04
Error	51	1.02	0.05	1.7E‐4	8.6E‐4	1.0E‐3	4.3E‐3

All factors and interactions were considered fixed effects. The residual was used as the error term for all tested effects. Mean squares (MS) are presented.

^a^ Degrees of freedom.

^b^ Significant at the probability level of 0.01.

*Sesamia nonagrioides* infestation or *Fusarium verticillioides* inoculation alone significantly reduced DFAT content, but the presence of both biotic factors significantly increased DFAT compared to control conditions, showing a synergistic effect on maize (Fig. 3). In non‐inoculated plants, *Sesamia nonagrioides* infestation led to a general reduction in FA and diferulate content compared to control plants. In contrast, *Sesamia nonagrioides* infestation in plants grown from seeds inoculated with *Fusarium verticillioides* resulted in opposite changes, increases in FA and diferulate contents (Fig. 3).

**Fig. 3:**
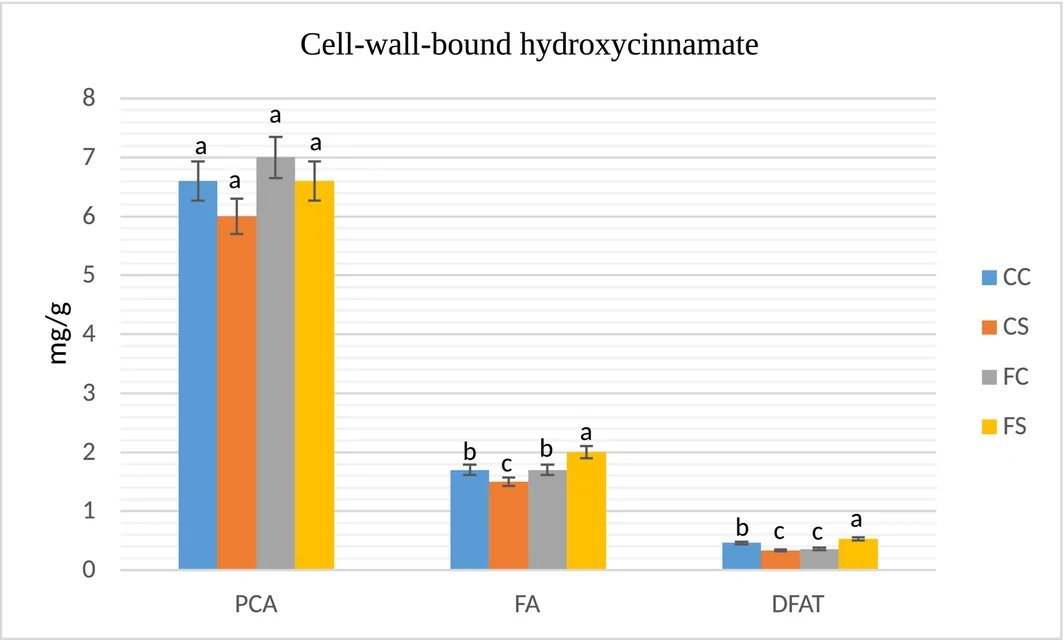
Concentration of *p*‐coumarate (*p*CA), ferulate (FA) and total diferulates in stem pith of seven maize inbred lines evaluated under four treatments: control (CC), infestation with *Sesamia nonagrioides* (CS), inoculation with *Fusarium verticillioides* (FC) and plants inoculated with *Fusarium verticillioides* and subsequently infested with *Sesamia nonagrioides* (FS). Vertical bars represent standard errors. Different letters indicate significant differences among treatments for each trait at the 0.05 probability level.

However, some individual inbreds had a differential performance as the genotype × treatment interaction was significant for all hydroxycinnamate traits (Table 4). The study of the responses of each inbred on hydroxycinnamate content to *Fusarium verticillioides* (FC *versus* CC), *Sesamia nonagrioides* without previous infection by *Fusarium verticillioides* (CS *versus* CC) and to *Sesamia nonagrioides* on plants previously infected by *Fusarium verticillioides* (FS *versus* FC) is shown in Table S2 and Fig. S2.

Variation among genotypes for tunnel length made by *Sesamia nonagrioides* was significant (*P* < 0.05), and it would be of great value to ascertain the role of each constitutive and induced cell‐wall‐bound phenolic compound on genotype resistance (Fig. S3). A multiple regression model incorporating both constitutive and induced cell wall traits explained 95% of the genetic variation for relative tunnel length under different scenarios (seeds previously infected and non‐infected by *Fusarium verticillioides*) (Table 5).

**Table 5:** Stepwise multiple linear regression model and equation for relative tunnel length of 14 *Fusarium verticillioides* treatment—genotype combinations, based on pre‐existing and induced (i_) levels by *Sesamia nonagrioides* attack for cell wall components. Pre‐existing levels refer to those observed in each of the seven inbreds under non‐infested conditions for each inoculation treatment (seed inoculation with *Fusarium verticillioides* and no inoculation), whereas induced levels represent the difference between induced and pre‐existing levels for each *Fusarium verticillioides* treatment—genotype combination.

tunnel length	partial *R* ^2^	*R* ^2^
S	0.25	0.25
i_G	0.34	0.58
FA	0.15	0.73
*p*CA	0.18	0.91
DFA 8‐*O*‐4	0.04	0.95
Model	−1.44 * S + 1.19 * i_G + 1.45 * FA – 0.95 * *p*CA – 0.23 * DFA 8‐*O*‐4	

Partial *R* ^2^: Percentage of variance explained by each character. *R* ^2^: Accumulative percentage of variance explained by adding independent variables. S: pre‐existing level of the S lignin subunit (%); i_G: induced level of the G lignin subunit (%); FA: pre‐existing level of ferulate; *p*CA: pre‐existing level of *p*‐coumarate concentration; DFA 8‐*O*‐4: pre‐existing level of DFA 8‐*O*‐4 concentration.

The final model retained five variables: the constitutive levels of the lignin S subunit, FA, *p*CA and DFA 8‐*O*‐4, along with the induced change in the G subunit (i_G). Constitutive levels of the S subunit, *p*CA and DFA 8‐*O*‐4 were negatively correlated with relative tunnel length. Conversely, higher constitutive levels of FA and stronger induction of the G subunit were positively correlated with relative tunnel length. These results highlight that both basal and induced lignin composition contributed to maize resistance against *Sesamia nonagrioides*.

## DISCUSSION

Accumulation of *p*CA, FA, DFAs and lignin represents a key mechanism of structural defence by reinforcing the cell wall matrix (Santiago *et al*. 2006a; Barros‐Rios *et al*. 2015; Gesteiro *et al*. 2021). However, cell‐wall phenolic compounds are typically quantified under control conditions as constitutive defences, and information on their induced levels following parasite attack remains scarce, despite extensive evidence showing that induced defences entail lower metabolic costs than constitutive ones (Santiago *et al*. 2006a, 2006b, 2011; Howe & Jander 2008; Rodríguez *et al*. 2021; Guo *et al*. 2023). However, the induction of a stress response implies that resistance mechanisms are activated only after contact with the stressor, potentially delaying the establishment of an effective response (Hilker & Schmülling 2019). This is particularly relevant given that some resistant maize displays a limited inducible response to attack, yet maintains high resistance levels through stronger constitutive defences (Guo *et al*. 2023). These findings suggest the existence of a continuum between defensive strategies, where highly resistant lines may invest in defence pre‐emptively, while susceptible or moderately resistant lines rely on a more plastic and adjustable defence activated upon damage.

Unlike previous studies that mainly evaluated maize cell wall defences against stem borers or fungal infection separately, the present work examined a combined and sequential interaction between *Fusarium verticillioides* seed inoculation and *Sesamia nonagrioides* infestation. This approach revealed that prior fungal inoculation can modify the biochemical cell wall response induced by insect attack, highlighting the importance of stress history in shaping maize structural defences.

The results obtained in this study provide a novel perspective on the complex interaction between constitutive, induced and priming‐modulated defences. As a first approach, the combined analysis of *Sesamia nonagrioides* infestation and *Fusarium verticillioides* infection showed that both factors—independently—trigger significant responses in the composition and accumulation of structural cell wall compounds. However, these responses were strongly modulated by the genotype. Moreover, the effects of both biotic factors on maize cell‐wall‐bound phenolic components are not additive, but synergistic. DFAT in the internode pith tissues decreased significantly in response to *Sesamia nonagrioides* or *Fusarium verticillioides* alone, but behaved in the opposite way when plants derived from *Fusarium*‐inoculated seeds were exposed to *Sesamia nonagrioides*. The initial reduction caused by *Fusarium verticillioides* appears to trigger a compensatory response after *Sesamia nonagrioides* attack, with a marked induction of diferulates, suggesting a mechanism similar to that described in other plant–microbe interactions (Martinez‐Medina *et al*. 2016; Erb & Reymond 2019; Wang *et al*. 2025). *Fusarium verticillioides* treatment also influenced the lignin composition response of plants to *Sesamia nonagrioides* attack. In the absence of *Fusarium verticillioides, Sesamia nonagrioides* infestation significantly affected only the proportion of S subunits, which decreased after insect attack. By contrast, in plants derived from *Fusarium verticillioides*‐inoculated seeds, infestation was associated with broader changes in lignin composition, including a significant decrease in S subunits and in the S/G ratio, together with an increase in G subunits. This differential response may be related to the contrasting functions of lignin subunits. Lignin G contributes to the rigidity and reinforcement of vascular tissues such as the xylem, promoting efficient water transport by waterproofing and stabilizing the cell walls. In contrast, lignins with a higher proportion of S subunits display a less condensed structure, which may facilitate more dynamic responses to biotic attacks (Zheng *et al*. 2017; Ma *et al*. 2018; Wang *et al*. 2024).

These results highlight the importance of considering possible pre‐existing conditions, such as the presence of *Fusarium verticillioides* infecting the seeds, which is a prevalent scenario in temperate regions (Wilke *et al*. 2007; Santiago *et al*. 2020), when the response to a parasite is studied. The previous exposure history of the plant appears to modify cell wall‐associated defence responses in certain maize genotypes. Initially, this alteration reduces investment in costly structural compounds, thereby enabling a more flexible and robust response to a subsequent attack. Consequently, the balance between constitutive and induced defences appears to be highly dynamic, depending on genotype, ontogeny and the history of exposure to multiple stresses (Boege & Marquis 2005; Hilker & Schmülling 2019). This flexibility has been identified as a critical factor in enhancing the efficiency of resource utilization under natural conditions, where attack events are stochastic and multiple biotic factors can coexist (Ballhorn *et al*. 2008; Moreira *et al*. 2014; Guo *et al*. 2023).

Once the induction of cell‐wall phenolic compounds by *Sesamia nonagrioides* attack has been demonstrated (Santiago & Malvar 2010; Santiago *et al*. 2013a; Gesteiro *et al*. 2021)—along with its dependence on maize genotype and prior infection by *Fusarium verticillioides*—it becomes essential to determine how these changes contribute to resistance against *Sesamia nonagrioides*. The multiple regression model indicated that the constitutive levels of the lignin S subunit, FA, *p*CA and DFA 8‐*O*‐4, together with the induced response of the lignin G subunit, explain up to 95% of the variability in damage caused by *Sesamia nonagrioides*, measured as relative tunnel length. These findings highlight that not only the absolute content of these compounds is crucial, but also their capacity for modulation upon attack—underscoring inducibility as a key functional trait, since such metabolic plasticity can be harnessed in breeding programs to select genotypes with efficient induced responses without compromising growth (Kumar *et al*. 2023).

In sum, these findings indicate that resistance to *Sesamia nonagrioides* does not rely solely on constitutive cell wall defences. Rather, it also depends on maize's capacity to dynamically coordinate cell wall metabolism under complex conditions, in which plant–insect–fungus interactions play a central role in shaping the defensive phenotype.

## AUTHOR CONTRIBUTIONS

NG: Supervision, formal analysis, investigation, writing—original draft, writing—review and editing. AB: Conceptualization, formal analysis, resources, supervision, funding acquisition. PL: Methodology, investigation. XCS: Project administration. RAM: Visualization, supervision. RS: Visualization, supervision, funding acquisition. All authors have read and approved the final version of the manuscript.

## FUNDING INFORMATION

This work was supported by projects PID2025‐169355OB‐I00 and PID2024‐157743OB‐I00, funded by MICIU/AEI/10.13039/501100011033 and FSE+.

MICIU refers to the Spanish Ministry of Science, Innovation and Universities (*Ministerio de Ciencia, Innovación y Universidades*), AEI to the Spanish State Research Agency (*Agencia Estatal de Investigación*), and FSE+ to the European Social Fund Plus (*Fondo Social Europeo Plus*). [Correction added on 15 September 2026, after first online publication: The funding information section has been added in this version.]

## CONFLICT OF INTEREST STATEMENT

The authors declare no conflict of interest.

## Supporting information


**Table S1.** ANOVA mean squares for Klason lignin (KL) and lignin subunits (S, G, H, S/G) in stem pith of seven maize inbreds under three comparisons: CC *versus* CS; CC *versus* FC; CS *versus* FS.
**Table S2.** ANOVA mean squares for cell wall–bound hydroxycinnamates in stem pith of seven maize inbreds under three comparisons: CC *versus* CS; CC *versus* FC; CS *versus* FS.
**Fig. S1.** KL content and lignin subunits (S, G, H) in seven maize inbreds under CC *versus* CS (A) and FC *versus* FS (B). Bars = SE. Different letters indicate significant differences (*P* < 0.05). Asterisks indicate CC *versus* FC differences.
**Fig. S2.** *p*CA, FA and total diferulates in seven maize inbreds under CC *versus* CS (A) and FC *versus* FS (B). Individual diferulate proportions are shown. Bars = SE. Different letters indicate significant differences (*P* < 0.05). Asterisks indicate CC *versus* FC differences.
**Fig. S3.** Mean tunnel length (± SE) of each inbred under non‐inoculated and *Fusarium*‐inoculated conditions. Dots and dashed lines show regression estimates (Table 5).

## Data Availability

The data that support the findings of this study are available on request from the corresponding author. The data are not publicly available due to privacy or ethical restrictions.
