# Supplementary material for: Fusarium verticillioides seed inoculation modifies maize cell wall responses to subsequent Sesamia nonagrioides infestation
Source: Plant Biol (Stuttg). 2026 Sep 10;28(7):2246–54. doi: 10.1111/plb.70284 (PMC13750340; doi:10.1111/plb.70284)
Supplement: Supplementary file 1 [file PLB-28-2246-s001.docx]

**Supplementary Table 1**. Mean squares of the analysis of variance of seven maize inbreds evaluated for Klason lignin (KL) content and percentages of syringyl (S), guaiacyl (G), p-hydroxyphenyl (H) subunits, and syringyl/guaiacyl (S/G) ratio in stem pith under three treatment comparisons: (i) control (CC) vs infested with Sesamia (CS); (ii) control (CC) vs inoculated with *Fusarium* (FC); and (iii) *Sesamia*-infested (CS) vs *Fusarium*-inoculated and subsequently infested with *Sesamia* (FS).

| Control (CC) vs infested with *Sesamia* (CS) | | | | | | |
| --- | --- | --- | --- | --- | --- | --- |
|  | DF^1^ | KL | S | G | H | S/G |
| Treatment | 1 | 2.40 | 24.47 | 8.44 | 6.96* | 0.07 |
| Genotype | 6 | 32.92** | 62.96** | 135.77** | 34.14** | 0.27** |
| Treatment*Genotype | 6 | 47.90** | 22.88* | 12.81 | 11.73** | 0.04 |
| Error | 25 | 8.87 | 7.30 | 9.66 | 1.52 | 0.03 |
| Control (CC) vs inoculated with *Fusarium* (FC) | | | | | | |
|  | DF^1^ | KL | S | G | H | S/G |
| Treatment | 1 | 7.92 | 1.01 | 1.66 | 4.04* | 4.8E-7 |
| Genotype | 6 | 96.95** | 109.73** | 168.81** | 43.24** | 0.40** |
| Treatment*Genotype | 6 | 17.03* | 14.58* | 22.30* | 9.63** | 0.06* |
| Error | 26 | 6.32 | 4.88 | 7.21 | 0.93 | 0.02 |
| *Sesamia* infestation (FC) vs inoculated with *Fusarium* and subsequently infested with *Sesamia* (FS) | | | | | | |
|  | DF^1^ | KL | S | G | H | S/G |
| Treatment | 1 | 2.26 | 39.50** | 67.52** | 2.64 | 0.21** |
| Genotype | 6 | 45.97** | 39.14** | 105.93** | 43.68** | 0.19** |
| Treatment*Genotype | 6 | 53.67** | 47.55** | 16.89* | 23.00** | 0.10** |
| Error | 26 | 6.24 | 3.13 | 6.67 | 2.46 | 0.01 |

^1^ Degrees of freedom.

*Significant at the probability level of 0.05.

**Significant at the probability level of 0.01.

**Supplementary Figure 1.** Klason lignin content and syringyl (S) subunits of seven maize inbred lines evaluated under four treatments: (**A**) control (CC) and infested with *Sesamia* (CS); and (**B**) inoculated with *Fusarium* without subsequent *Sesamia* infestation (FC) and inoculated with *Fusarium* and subsequently infested with *Sesamia* (FS). The percentage contribution of syringyl (S), guaiacyl (G), and p-hydroxyphenyl (H) subunits to lignin is also shown for each genotype × treatment combination. Vertical bars represent standard errors. Genotype × treatment combinations with different letters differ significantly at the 0.05 probability level. The asterisk indicates significant differences between non-inoculated and inoculated plants of the same inbred in the absence of infestation (CC vs FC).

**Supplementary Table 2.** Mean squares of the analysis of variance of seven maize inbreds evaluated for esterified hydroxycinnamates content in the cell wall, in stem pith under three treatment comparisons: (i) control (CC) vs infested with Sesamia (CS); (ii) control (CC) vs inoculated with Fusarium (FC); and (iii) Sesamia-infested (CS) vs Fusarium-inoculated and subsequently infested with Sesamia (FS).

| Control (CC) vs infested with *Sesamia* (CS) | | | | | | | |
| --- | --- | --- | --- | --- | --- | --- | --- |
|  | DF^1^ | *p*-coumarate | ferulate | DFA 5-5 | DFA 8-*O*-4 | DFA 8-5 | DFAT |
| Treatments | 1 | 2.87 | 0.37** | 1.2E-3* | 0.07** | 0.02** | 0.18** |
| Genotype | 6 | 37.56** | 1.22** | 2.0E-3** | 0.01** | 0.01** | 0.05** |
| Treat*Genotype | 6 | 4.19* | 0.06 | 6.8E-4** | 0.01** | 5.6E-3** | 0.03** |
| Error | 24 | 1.59 | 0.04 | 1.7E-4 | 9.0E-4 | 6.2E-4 | 3.4E-3 |
| Control (CC) vs inoculated with *Fusarium* (FC) | | | | | | | |
|  | DF^1^ | *p*-coumarate | ferulate | DFA 5-5 | DFA 8-*O*-4 | DFA 8-5 | DFAT |
| Treatments | 1 | 2.11 | 6.5E-4 | 2.2E-6 | 5.7E-2** | 5.1E-3* | 9.8E-2** |
| Genotype | 6 | 28.46** | 1.10** | 3.3E-3** | 9.5E-3** | 1.4E-2** | 5.1E-2** |
| Treat*Genotype | 6 | 2.33* | 0.15* | 1.0E-3** | 1.2E-2** | 7.6E-3** | 4.9E-2** |
| Error | 24 | 0.94 | 4.4E-2 | 1.3E-4 | 1.5E-3 | 9.8E-4 | 5.1E-3 |
| *Sesamia* infestation (FC) vs inoculated with *Fusarium* and subsequently infested with *Sesamia* (FS) | | | | | | | |
|  | DF^1^ | *p*-coumarate | ferulate | DFA 5-5 | DFA 8-*O*-4 | DFA 8-5 | DFAT |
| Treatments | 1 | 1.63 | 0.83** | 1.7E-3** | 0.13** | 0.02** | 0.31** |
| Genotype | 6 | 29.11** | 1.14** | 2.2E-3** | 0.01** | 0.01** | 0.04** |
| Treat*Genotype | 6 | 4.73** | 0.19** | 1.2E-3** | 0.02** | 0.01** | 0.08** |
| Error | 27 | 0.52 | 0.05 | 1.8E-4 | 8.3E-4 | 1.4E-3 | 5.2E-3 |

^1^ Degrees of freedom.

*Significant at the probability level of 0.05.

**Significant at the probability level of 0.01.

**Supplementary Figure 2.** Concentration of p-coumarate (pCA), ferulate (FA), and total diferulates of seven inbred lines evaluated under four treatments: (A) control (CC) and infested with Sesamia (CS); and (B) inoculated with Fusarium without subsequent Sesamia infestation (FC) and inoculated with Fusarium and subsequently infested with Sesamia (FS). The percentage contribution of each individual diferulate (DFA 8–5, DFA 8–O–4, DFA 5–5) to total diferulate content is also shown for each treatment-genotype combination. Vertical bars indicate standard errors, and genotype × treatment combinations with different letters differed significantly at the 0.05 probability level. The asterisk indicates significant differences between non-inoculated and inoculated plants of the same inbred in the absence of infestation (CC vs FC).

**Supplementary Figure 3**. Mean tunnel length (± se) for each maize inbred line under two treatments: No inoculation (blue) and inoculation with *Fusarium* (orange). Dots and dashed lines represent the values estimated by the multiple regression model shown in Table 5.
